# Supplementary material for: Chemical Composition and Flavor Characteristics of Cider Fermented with Saccharomyces cerevisiae and Non-Saccharomyces cerevisiae
Source: Foods. 2023 Sep 26;12(19):3565. doi: 10.3390/foods12193565 (PMC10572567; doi:10.3390/foods12193565)
Supplement: Supplementary file 1 [file foods-12-03565-s001.zip › foods-2597542-supplementary.pdf]

Supplementary Table S1 The volatile flavor compounds identified by GC/MS of apple juice and cider samples fermented with different non-*Saccharomyces* yeast

No.	Name	Formula	FAJ	Cider (μg/L)				
				Sc	Sc+Zb	Sc+Rm	Sc+Km	Sc+Dh
C01	2,3-Butanediol	$C_4H_{10}O_2$	nd	16.05 ±2.84a	24.41 ± 28.74a	36.15 ± 12.52a	16.70 ± 3.38a	39.08 ± 6.83a
C02	2-Furanmethanol	$C_5H_6O_2$	nd	nd	nd	3.91 ± 5.53	nd	nd
C03	1-Butanol, 3-methyl-	$C_5H_{12}O$	20.22 ± 0.57b	1954.68 ± 412.61a	1860.53 ± 464.12a	1492.47 ± 189.52a	1292.73 ± 35.12a	1510.91 ± 243.64a
C04	3-Hexen-1-ol, (Z)-	$C_6H_{12}O$	8.94 ± 2.04	nd	nd	nd	nd	nd
C05	Phenylethyl alcohol	$C_8H_{10}O$	nd	561.18 ± 40.14a	614.18 ± 213.97a	609.01 ± 187.79a	488.65 ± 277.71a	782.68 ± 165.47a
C06	Cyclohexanol, 5-methyl-2-(1-methylethyl)-	$C_{10}H_{20}O$	10.49 ± 1.24	nd	nd	nd	nd	nd
C07	1-Dodecanol	$C_{12}H_{26}O$	nd	nd	nd	nd	3.00 ± 4.25	nd
C08	5,9-Undecadien-2-ol, 6,10-dimethyl-	$C_{13}H_{24}O$	nd	nd	nd	29.21 ± 9.79	nd	nd
C09	2,6,10-Dodecatrien-1-ol, 3,7,11-trimethyl-	$C_{15}H_{26}O$	nd	nd	9.12 ± 12.89a	nd	nd	27.30 ± 20.36a
	Alcohols		39.65 ±1.88b	2531.91 ±446.04a	2508.24 ± 491.84a	1801.08 ± 248.36a	2359.97 ± 304.51a	2170.75 ± 401.24a
C1	Hexanal	$C_6H_{12}O$	169.80 ± 1.39	nd	nd	nd	nd	nd

0								
C11	Furfural	$C_5H_4O_2$	143.77 ± 9.57	nd	nd	nd	9.28 ±	nd
C12	Benzaldehyde, 2,5-dimethyl-	$C_9H_{10}O$	nd	32.25 ± 45.61a	18.51 ± 26.18a	49.09 ± 69.42a	24.42 ± 34.54a	nd
	Aldehydes		313.57 ± 10.62a	32.25 ± 45.61b	18.51 ± 26.18b	33.70 ± 28.00b	nd	49.09 ± 69.42b
C13	Butanoic acid, 2-methyl-, ethyl ester	$C_7H_{14}O_2$	75.83 ± 0.96	nd	nd	nd	nd	nd
C14	Butanoic acid, 3-methyl-, ethyl ester	$C_7H_{14}O_2$	129.09 ± 0.95	nd	nd	nd	nd	nd
C15	Acetic acid, isoamyl ester	$C_7H_{14}O_2$	897.21 ± 10.98a	73.87 ±1.37b	72.30 ±7.21b	33.14 ± 8.25c	50.62 ± 13.49bc	67.47 ± 20.07b
C16	Propanoic acid, 2-hydroxy-, 2-methylpropyl ester	$C_7H_{14}O_3$	nd	nd	nd	7.82 ± 5.68a	nd	4.43 ± 6.27a
C17	4-Hexen-1-ol, acetate	$C_8H_{14}O_2$	65.72 ± 8.51	nd	nd	nd	nd	nd
C18	Hexanoic acid, ethyl ester	$C_8H_{16}O_2$	nd	190.52 ± 40.88a	124.91 ± 88.90a	170.39 ± 22.25a	108.20 ± 81.02a	163.04 ± 68.91a
C19	Acetic acid, hexyl ester	$C_8H_{16}O_2$	89.92 ± 6.90	nd	nd	nd	nd	nd
C20	Acetic acid, 2-phenylethyl ester	$C_{10}H_{12}O_2$	nd	86.51 ± 16.89a	155.90 ± 84.81a	104.32 ± 47.93a	151.46 ± 86.91a	147.74 ± 24.55a
C21	Octanoic acid, ethyl ester	$C_{10}H_{20}O_2$	nd	829.30 ± 333.98a	848.25 ±98.09a	853.75 ± 60.95a	641.14 ± 188.03b	1345.22 ± 476.32a

C22	9-Decenoic acid, ethyl ester	$C_{12}H_{22}O_2$	nd	51.49 ± 18.59a	43.40 ± 43.98a	30.80 ± 43.55a	10.85 ± 15.34a	78.08 ± 27.72a
C23	Decanoic acid, ethyl ester	$C_{12}H_{24}O_2$	nd	683.19 ± 68.36a	665.49 ± 104.18a	690.20 ± 119.92a	579.15 ± 204.16a	845.10 ± 66.20a
C24	4-Ethylbenzoic acid, 2-butyl ester	$C_{13}H_{18}O_2$	nd	42.93 ± 4.99b	46.41 ± 15.34b	51.73 ± 14.13a	34.67 ± 6.61b	80.06 ± 27.07a
C25	Acetic acid, [4-(1,1-dimethylethyl)phenoxy]-, methyl ester	$C_{13}H_{18}O_3$	nd	nd	166.72 ± 235.78a	90.07 ± 127.38a	nd	nd
C26	Tridecanoic acid, 3-hydroxy-, ethyl ester	$C_{15}H_{30}O_3$	nd	nd	nd	10.29 ± 2.63a	3.82 ± 5.40b	12.55 ± 1.83a
C27	Nerolidyl acetate	$C_{17}H_{28}O_2$	nd	nd	nd	nd	1.97 ± 2.78	nd
C28	Hexadecanoic acid, ethyl ester	$C_{18}H_{36}O_2$	nd	nd	nd	64.06 ±90.60a	22.75 ± 32.18a	nd
C29	Succinic acid, hept-2-yl oct-1-en-3-yl ester	$C_{19}H_{34}O_4$	nd	nd	nd	6.02 ± 4.49a	2.83 ± 4.00a	nd
C30	Isopropyl palmitate	$C_{19}H_{38}O_2$	nd	8.68 ± 12.28a	131.29 ± 185.67a	8.08 ± 11.43a	nd	nd
C31	1,2-Benzenedicarboxylic acid, butyl octyl ester	$C_{20}H_{30}O_4$	nd	nd	nd	nd	35.83 ± 50.67	nd
C32	Benzoic acid, 2-formyl-4,6-dimethoxy-, 8,8-dimethoxyoct-2-yl	$C_{20}H_{30}O_7$	nd	4.46 ± 6.31	nd	nd	nd	nd

	ester							
C33	Decanoic acid, tetradecyl ester	$C_{24}H_{48}O_2$	nd	nd	nd	nd	nd	150.45 ± 212.76
	Esters		1257.76 ± 4.28d	1970.95 ± 448.70bc	2254.68 ± 321.71ab	1643.29 ± 411.02bc	2894.15 ± 346.26a	2120.68 ± 282.80b
C34	Acetic acid	$C_2H_4O_2$	nd	36.33 ± 6.22b	64.99 ± 33.35ab	71.96 ± 28.79ab	44.19 ± 6.50b	93.69 ± 10.43a
C35	Hexanoic acid	$C_6H_{12}O_2$	nd	158.21 ± 48.94b	204.63 ± 15.30ab	179.67 ± 20.69b	157.02 ± 68.56b	276.99 ± 43.38a
C36	Octanoic acid	$C_8H_{16}O_2$	nd	633.88 ± 14.41b	863.37 ± 299.93ab	903.24 ± 231.02ab	838.51 ± 405.44ab	1216.82 ± 207.03a
C37	9-Decenoic acid	$C_{10}H_{18}O_2$	nd	24.69 ± 17.49a	32.93 ± 31.28a	42.00 ± 18.40a	nd	62.72 ± 19.75a
C38	n-Decanoic acid	$C_{10}H_{20}O_2$	0.42 ± 0.59c	756.94 ± 42.87b	967.74 ± 284.78ab	951.23 ± 229.16ab	883.20 ± 540.30ab	1485.78 ± 192.57a
C39	10-Hydroxydecanoic acid	$C_{10}H_{20}O_3$	nd	nd	nd	nd	3.01 ± 4.26	nd
C40	Dodecanoic acid	$C_{12}H_{24}O_2$	nd	194.62 ± 28.45a	242.76 ± 40.87a	228.68 ± 35.16a	149.53 ± 115.25a	235.03 ± 166.23a
C41	n-Hexadecanoic acid	$C_{16}H_{32}O_2$	nd	nd	16.85 ± 23.83a	180.91 ± 237.56a	19.78 ± 14.71a	16.76 ± 12.02a
C42	Oleic Acid	$C_{18}H_{34}O_2$	nd	nd	nd	nd	22.37 ± 18.61	nd
	Acids		0.42 ± 0.59c	1804.68 ±	2393.27 ±	2117.61 ±	3387.78 ±	2557.69 ±

				123.56b	630.46ab	1162.74ab	377.85a	693.43ab
C43	2-Buten-1-one, 1-(2,6,6-trimethyl-1,3-cyclohexadien-1-yl)-, (E)-	$C_{13}H_{18}O$	5.97 ± 0.58	nd	nd	nd	nd	nd
	Ketone		5.97 ± 0.58	nd	nd	nd	nd	nd
C44	3-Allyl-6-methoxyphenol	$C_{10}H_{12}O_2$	nd	nd	4.62 ± 6.54b	14.69 ± 4.10a	12.79 ± 5.61b	nd
C45	2,4-bis(1,1-dimethylethyl)-phenol	$C_{14}H_{22}O$	9.60 ± 0.43b	94.88 ± 29.39ab	139.97 ± 73.96a	116.68 ± 53.11ab	120.66 ± 26.71ab	144.21 ± 72.50a
C46	2,4-bis(1,1-dimethylethyl)-5-methylphenol	$C_{15}H_{24}O$	nd	nd	4.14 ± 5.85	nd	nd	nd
	Phenols		9.60 ± 0.43b	94.88 ± 29.39ab	148.73 ± 73.96a	133.45 ± 26.71ab	144.21 ± 72.50a	131.37 ± 53.11ab
C47	p-Xylene	C_8H_{10}	nd	nd	42.38 ± 13.51a	nd	14.23 ± 10.28b	nd
C48	o-Xylene	C_8H_{10}	nd	nd	nd	nd	4.69 ± 6.63	nd
C49	Benzene, 1,2,4-trimethyl-	C_9H_{12}	nd	30.81 ± 10.22ab	45.47 ± 9.47a	24.97 ± 9.25b	34.83 ± 7.00ab	22.41 ± 6.74b
	Benzenes		nd	30.81 ± 10.22bc	87.85 ± 19.05a	53.75 ± 10.87b	22.41 ± 6.74c	24.97 ± 9.25c
C50	1-Octene, 3-(methoxymethoxy)-	$C_{10}H_{20}O_2$	nd	nd	5.28 ± 7.46a	4.25 ± 6.01a	nd	8.82 ± 6.24a
	Alkene		nd	nd	5.28 ± 7.46a	nd	8.82 ± 6.24a	4.25 ± 6.01a
	Total		1626.97 ±	6465.48 ±	7416.57 ±	5782.89 ±	8817.34 ±	7058.80 ±

	8.80c	502.14ab	1102.77ab	1814.94b	156.60ab	1407.18ab

Results represent the mean ± SD for three independent experiments. Mean values in the same row with the same letters are not significantly different at the 95% confidence level (Duncan's test). ND = not detected. Different letters represent significantly difference at the 95% percent confidence level (Duncan's test). Sc, *S. cerevisiae* monoculture. Sc+Zb, *S. cerevisiae* and *Z. bailii* co-inoculation. Sc+Rm, *S. cerevisiae* and *R. mucilaginosa* co-inoculation. Sc+Km, *S. cerevisiae* and *K. marxianus* co-inoculation. Sc+Dh, *S. cerevisiae* and *D. hansenii* co-inoculation. FAJ, apple juice.

Supplementary Table S2 Odor-activity valus (OVA) of apple juice anf cider samples fermented with different non-*Saccharomyces* yeast

No.	Name	Odor	Threshold value（μg/L）	FAJ	OVA（μg/L）				
					Sc	Sc+Zb	Sc+Rm	Sc+Km	Sc+Dh
	Alcohols								
C01	2,3-Butanediol	Butter, cheese aroma	668	nf	0.02 ± 0.00a	0.04 ± 0.04a	0.05 ± 0.02a	0.03 ± 0.01a	0.06 ± 0.01a
C02	2-Furanmethanol	Mildly irritating	8000	nf	nf	nf	nf	nf	nf
C03	1-Butanol, 3-methyl-	Mild odor	1.7	11.89 ± 0.34b	1149.81 ± 242.71a	1094.43 ± 580.27a	877.93 ± 111.48a	760.43 ± 20.66a	888.77 ± 143.32a
C04	3-Hexen-1-ol, (Z)-	Powerful grassy-green odor	0.013	687.87 ± 157.28	nf	nf	nf	nf	nf
C05	Phenylethyl Alcohol	floral odor of roses	100000	nf	0.01 ± 0.01a	0.01 ± 0.00a	0.01 ± 0.00a	0.00a	0.01 ± 0.00a
C06	Cyclohexanol, 5-methyl-2-(1-methylethyl)-	Peppermint	nf	nf	nf	nf	nf	nf	nf
C07	1-Dodecanol	Typical fatty alcohol sweet	1.2	nf	nf	nf	nf	2.50 ± 3.54	nf

C08	5,9-Unfecadien-2-ol, 6,10-dimethyl-	nf	nf	nf	nf	nf	nf	nf	nf
C09	2,6,10-Dodecatrien-1-ol, 3,7,11-trimethyl-	nf	nf	nf	nf	nf	nf	nf	nf
	Aldehydes								
C10	Hexanal	Pungent	0.28	606.41 ± 4.97	nf	nf	nf	nf	nf
C11	Furfural	Almonf-like	2.8	51.35 ± 3.42	nf	nf	nf	nf	nf
C12	Benzaldehyde, 2,5-dimethyl-	nf	nf	nf	nf	nf	nf	nf	nf
	Esters								
C13	Butanoic acid, 2-methyl-, ethyl ester	Apple, strawberry, berry, sweet, cider, anise	1	75.83 ± 0.96	nf	nf	nf	nf	nf
C14	Butanoic acid, 3-methyl-, ethyl ester	Sweet fruit, anise, floral	3	43.03 ± 0.32	nf	nf	nf	nf	nf
C15	Acetic acid, isoamyl ester	Banana-like	30	29.91 ± 0.37a	2.46 ± 0.05ab	2.41 ± 0.24ab	2.25 ± 0.67ab	1.10 ± 0.28b	1.69 ± 0.45ab
C16	Propanoic acid, 2-hydroxy-, 2-methylpropyl ester	nf	nf	nf	nf	nf	nf	nf	nf
C1	4-Hexen-1-ol,	nf	nf	nf	nf	nf	nf	nf	nf

7	acetate								
C18	Hexanoic acid, ethyl ester	Green apple, fennel flavor	14	nf	13.61 ± 2.92a	8.92 ± 6.33a	12.17 ± 1.59a	7.73 ± 5.79a	11.65 ± 4.92a
C19	Acetic acid, hexyl ester	Fruity aromas of pear anf cherry	670	0.13 ± 0.01	nf	nf	nf	nf	nf
C20	Acetic acid, 2-phenylethyl ester	Sweet, honey	6.7	nf	12.91 ± 2.52a	23.27 ± 12.56a	15.57 ± 7.15a	22.61 ± 12.97a	22.05 ± 3.66a
C21	Octanoic acid, ethyl ester	Waxy, sweet	40	nf	20.73 ± 8.35ab	21.21 ±2.45ab	21.34 ± 1.52ab	16.03 ± 4.70b	33.63 ± 11.91a
C22	9-Decenoic acid, ethyl ester	nf	nf	nf	nf	nf	nf	nf	nf
C23	Decanoic acid, ethyl ester	Rummy with a creamy, floral nuance	2	nf	341.60 ± 34.18a	332.75 ± 52.09a	345.10 ± 59.96a	289.58 ± 102.08a	422.55 ± 33.10a
C24	4-Ethylbenzoic acid, 2-butyl ester	nf	nf	nf	nf	nf	nf	nf	nf
C25	Acetic acid, [4-(1,1-dimethylethyl)phenoxy]-, methyl ester	nf	nf	nf	nf	nf	nf	nf	nf

C26	Tridecanoic acid, 3-hydroxy-, ethyl ester	nf	nf	nf	nf	nf	nf	nf	nf
C27	Nerolidyl acetate	nf	nf	nf	nf	nf	nf	nf	nf
C28	Hexadecanoic acid, ethyl ester	nf	nf	nf	nf	nf	nf	nf	nf
C29	Succinic acid, hept-2-yl oct-1-en-3-yl ester	nf	nf	nf	nf	nf	nf	nf	nf
C30	Isopropyl palmitate	Almost odorless	nf	nf	nf	nf	nf	nf	nf
C31	1,2-Benzenedicarboxylic acid, butyl octyl ester	nf	nf	nf	nf	nf	nf	nf	nf
C32	Benzoic acid, 2-formyl-4,6-dimethoxy-, 8,8-dimethoxyoct-2-yl ester	nf	nf	nf	nf	nf	nf	nf	nf
C33	Decanoic acid, tetradecyl ester	nf	nf	nf	nf	nf	nf	nf	nf
	Acids								
C34	Acetic acid	Sharp pungent sour vinegar	13	nf	2.79 ± 0.48ab	5.00 ± 2.57b	5.54 ±	3.40 ± 0.50b	7.21 ± 0.80a

							2.21ab		
C35	Hexanoic acid	Sour fatty sweat cheese	4.8	nf	32.96 ± 10.20ab	42.63 ± 3.19b	37.43 ± 4.31ab	32.71 ± 14.28b	57.71 ± 9.06a
C36	Octanoic acid	Vegetable cheesy	5.1	nf	124.29 ± 2.83a	169.29 ± 58.81a	177.11 ± 45.30a	164.41 ± 79.50a	238.59 ± 40.59a
C37	9-Decenoic acid	nf	nf	nf	nf	nf	nf	nf	nf
C38	n-Decanoic acid	Pleasant rancid sour fatty citrus	90	nf	8.41 ± 0.48a	10.75 ± 3.16a	10.57 ± 2.55a	9.81 ± 6.00a	16.51 ± 2.14a
C39	10-Hydroxydecanoic acid	nf	nf	nf	nf	nf	nf	nf	nf
C40	Dodecanoic acid	Mild fatty coconut bay oil	4	nf	48.66 ± 7.11a	43.39 ± 31.74a	57.17 ± 8.79a	37.38 ± 28.81a	58.76 ± 41.56a
C41	n-Hexadecanoic acid	Waxy, creamy, fatty	nf	nf	nf	nf	nf	nf	nf
C42	Oleic Acid	nf	nf	nf	nf	nf	nf	nf	nf

	Ketone								
C43	2-Buten-1-one, 1-(2,6,6-trimethyl-1,3-cyclohexadien-1-yl)-, (E)-	nf	nf	nf	nf	nf	nf	nf	nf
	Phenols								
C44	3-Allyl-6-methoxyphenol	nf	nf	nf	nf	nf	nf	nf	nf
C45	2,4-bis(1,1-dimethylethyl)-phenol	nf	nf	nf	nf	nf	nf	nf	nf
C46	2,4-bis(1,1-dimethylethyl)-5-methylphenol	nf	nf	nf	nf	nf	nf	nf	nf
	Benzenes								
C47	p-Xylene	Like benzene; characteristic aromatic.	58	nf	nf	0.73 ± 0.23	nf	0.25 ± 0.18	nf
C48	o-Xylene	Benzene-like; characteristic aromatic.	380	nf	nf	nf	nf	0.01 ± 0.02	nf
C49	Benzene, 1,2,4-trimethyl-	Distinctive, aromatic odor	120	nf	0.26 ± 0.09ab	0.38 ± 0.08ab	0.21 ± 0.08a	0.29 ± 0.06ab	0.19 ± 0.06b
	Alkene								

C50	1-Octene, 3-(methoxymethoxy)-	nf	nf	nf	nf	nf	nf	nf	nf

Results represent the mean ± SD for three infepenfent experiments. Mean values in the same row with the same letters are not significantly different at the 95% confidence level (Duncan's test). Thresholds refer to thresholds in water and air. nf = no threshold was found. Different letters represent significantly difference at the 95% percent confidence level (Duncan's test). Sc, *S. cerevisiae* monoculture. Sc+Zb, *S. cerevisiae* anf *Z. bailii* co-inoculation. Sc+Rm, *S. cerevisiae* anf *R. mucilaginosa* co-inoculation. Sc+Km, *S. cerevisiae* anf *K. marxianus* co-inoculation. Sc+Dh, *S. cerevisiae* anf *D. hansenii* co-inoculation. FAJ, apple juice.

Supplementary Table S3 GC-IMS global area set integration parameters obtained from apple juice and cider samples fermented with different non-*Saccharomyces* yeast

Count	Compound	CAS#	Formula	MW	RI	Rt [sec]	Dt [a.u.]	Comment
1	Acetic acid	C64197	$C_2H_4O_2$	60.1	1496.3	1182.294	1.15026	
2	Octanoic acid ethyl ester	C106321	$C_{10}H_{20}O_2$	172.3	1450.0	1078.527	1.48342	Monomer
3	Octanoic acid ethyl ester	C106321	$C_{10}H_{20}O_2$	172.3	1448.7	1075.747	2.03352	Dimer
4	Ethyl lactate	C97643	$C_5H_{10}O_3$	118.1	1351.0	886.122	1.54168	
5	Ethyl hexanoate	C123660	$C_8H_{16}O_2$	144.2	1235.0	706.102	1.80106	
6	3-Methyl-1-butanol	C123513	$C_5H_{12}O$	88.1	1210.7	673.855	1.50511	
7	Isoamyl acetate	C123922	$C_7H_{14}O_2$	130.2	1123.5	521.833	1.75012	
8	2-Methyl-1-propanol	C78831	$C_4H_{10}O$	74.1	1097.9	480.847	1.37251	
9	Ethyl 3-methylbutanoate	C108645	$C_7H_{14}O_2$	130.2	1065.5	440.546	1.26456	Monomer
10	Ethyl 3-methylbutanoate	C108645	$C_7H_{14}O_2$	130.2	1064.3	439.125	1.65941	Dimer
11	Ethyl 2-methylbutanoate	C7452791	$C_7H_{14}O_2$	130.2	1048.0	420.413	1.656	
12	Ethyl butanoate	C105544	$C_6H_{12}O_2$	116.2	1036.7	407.86	1.56134	
13	2-Methylpropyl acetate	C110190	$C_6H_{12}O_2$	116.2	1013.7	383.464	1.61933	
14	2-Pentanone	C107879	$C_5H_{10}O$	86.1	981.2	354.659	1.3786	
15	Ethyl acetate	C141786	$C_4H_8O_2$	88.1	872.9	283.419	1.34468	
16	Ethanol	C64175	C_2H_6O	46.1	918.0	311.168	1.1329	
17	Acetone	C67641	C_3H_6O	58.1	814.5	251.17	1.11831	
18	Furfural	C98011	$C_5H_4O_2$	96.1	1485.2	1156.53	1.08772	Monomer
19	Furfural	C98011	$C_5H_4O_2$	96.1	1488.7	1164.774	1.34056	Dimer
20	(Z)-3-hexenyl acetate	C3681718	$C_8H_{14}O_2$	142.2	1323.6	839.039	1.82042	
21	Ethyl propanoate	C105373	$C_5H_{10}O_2$	102.1	959.0	338.739	1.45712	
22	Hexanal	C66251	$C_6H_{12}O$	100.2	1087.9	467.8	1.56455	

23	Butan-1-ol	C71363	$C_4H_{10}O$	74.1	1148.7	565.785	1.38459	
24	Methyl hexanoate	C106707	$C_7H_{14}O_2$	130.2	1183.6	632.58	1.68889	
25	(Z)-3-hexenol	C928961	$C_6H_{12}O$	100.2	1395.2	967.324	1.22876	Monomer
26	(Z)-3-hexenol	C928961	$C_6H_{12}O$	100.2	1396.5	969.772	1.51253	Dimer
27	1-Hexanol	C111273	$C_6H_{14}O$	102.2	1362.7	906.929	1.64043	Monomer
28	1-Hexanol	C111273	$C_6H_{14}O$	102.2	1361.8	905.297	1.99814	Dimer
29	3-Pentanone	C96220	$C_5H_{10}O$	86.1	978.6	352.752	1.35687	

Supplementary Table S4 Physicochemical parameters of apple juice and cider samples fermented with different non-*Saccharomyces* yeast

Samples	FAJ	Ciders				
		Sc	Sc+Zb	Sc+Rm	Sc+Km	Sc+Dh
Tartaric acid (g/L)	1.40 ± 0.02a	0.52 ± 0.02b	0.52 ± 0.04b	0.33 ± 0.04c	0.34 ± 0.02c	0.28 ± 0.11c
Malic acid (g/L)	1.14 ± 0.02a	0.68 ± 0.01b	0.46 ± 0.06c	0.11 ± 0.00d	0.17 ± 0.02e	0.18 ± 0.01e
Acetic acid (g/L)	2.18 ± 0.04a	2.32 ± 0.05a	2.66 ± 0.05ab	3.04 ± 0.13b	3.03 ± 0.08b	2.44 ± 0.56a
Citric acid (g/L)	0.38 ± 0.00a	0.46 ± 0.03b	ND	0.49 ± 0.02b	ND	0.43 ± 0.06ab
Succinic acid (g/L)	ND	0.38 ± 0.13a	0.27 ± 0.09a	0.17 ± 0.02ab	0.23 ± 0.05ab	0.21 ± 0.01ab

Results represent the mean ± SD for three independent experiments. Different letters represent significantly difference at the 95% confidence level (Duncan's test). ND = not detected. TA = total acid (expressed as percentage of tartaric acid). Sc, *S. cerevisiae* monoculture. Sc+Zb, *S. cerevisiae* and *Z. bailii* co-inoculation. Sc+Rm, *S. cerevisiae* and *R. mucilaginosa* co-inoculation. Sc+Km, *S. cerevisiae* and *K. marxianus* co-inoculation. Sc+Dh, *S. cerevisiae* and *D. hansenii* co-inoculation. FAJ, apple juice.
